# The complete mitochondrial genome of *Cerithidea tonkiniana* (Mabille, 1887) in Guangxi, China

**DOI:** 10.1080/23802359.2021.2006813

**Published:** 2022-04-19

**Authors:** Shangchen Yang, Zhangwen Deng

**Affiliations:** a College of Life Sciences, Zhejiang University, Hangzhou, China; b Guangxi Zhuang Autonomous Region Forest Inventory and Planning Institute, Nanning, China

**Keywords:** *Cerithidea tonkiniana*, mitochondrial genome, phylogenetic analysis, Cerithioidea

## Abstract

*Cerithidea tonkiniana* is an amphibious gastropod mollusk that lives in brackish water habitats. In this study, the complete mitochondrial genome of *Cerithidea tonkiniana* collected from Guangxi, China was assembled for the first time based on the next generation sequencing. The length of this mitogenome is 15,617 base pair with a slightly biased AT content (63.1％). This circular genome contains 13 proterin-coding genes, 22 tRNA genes, and 2 rRNA genes. Phylogenetic analysis using the 10 Cerithioidea species showed that the *C. tonkiniana* is closely related with *Cerithidea sinensis* and *Cerithidea obtuse*, forming the Potamididae cluster.

The *Cerithidea tonkiniana* is one of the 29 extant gastropod mollusks in the Potamididae, which consists of snails that are closely associated with mangrove forests (Reid et al. 2008). The amphibious *C. tonkiniana* lives mainly on mangrove trunks and branches, rotten wood and mud under mangroves, and is widely distributed in China, Japan and Vietnam (Reid 2014). Because of its specific brackish water habitat and amphibious lifestyle, *C. tonkiniana* is a potential model species to study the mechanisms of adaptive evolution and water-to-land transition.

Unique characteristics including high copy number, usually maternal inheritance and stable evolutionary rate, make mitochondrial DNA particularly suitable for evolutionary studies (Stoneking and Soodyall 1996). Complete mitochondrial genomes have been widely and successfully used in population genetics, phylogenetic relationship, and phylogeography. Here we reported the complete mitochondrial genome of *C. tonkiniana* and the phylogenetic relationships among its closely related species, providing valuable reference and resources for further research on *C. tonkiniana.*

One *C. tonkiniana* individual was collected from Guangxi Maoweihai Mangrove Nature Reserve, Beihai, Guangxi, China (21°40′N, 108°30′E). The dissected head foot tissues were used for total genomic DNA isolation. A sample of ∼1μg genomic DNA was sheared into fragments with 200–800 base pair (bp) for construction of a paired-end DNA library with insert size of 300 bp for Illumina sequencing. This snail specimen was deposited at the Guangxi Forest Inventory & Planning Institute (http://www.gxforestry.com/, 260810771@qq.com) under the voucher number SSDCF20201027. NOVOPlasty software (Dierckxsens et al. 2017) was employed to assemble the mitochondrial genome, using the COI sequence of the *Cerithidea sinensis* (Xu et al. 2019) as the *seed input*. MITOS (http://mitos2.bioinf.uni-leipzig.de/) was used for gene annotation.

We obtained 123 Mb raw reads in total, and the aligned reads number was 122,726 by using NOVOPlasty software, with the average organelle coverage reaching 786 X. The final complete mitogenome of *C. tonkiniana* is 15,617 bp. The base composition of this genome was 29.3％ of A, 33.8％ of T, 18. 5％ of C and 18.4％ of G, showing an obvious AT bias (63.1％) like mitochondrial genomes from other Potamididae species. We further characterized the detailed information of this mitochondrial genome to facilitate the use of this genome by other studies. This genome contained 37 genes in total, including 13 protein-coding genes (PSGs), 22 transfer RNA (tRNA) genes and 2 ribosomal RNA (rRNA) genes. The gene composition and organization was highly similar with those of *Cerithidea sinensis* and *Cerithidea obtuse* (Xu et al. 2019), indicating the conserved gene structure among animals in Potamididae family.

The phylogenetic relationships between *C. tonkiniana* and other snails of Cerithioidea were further explored by using 10 whole mitogenomes, and the genome of *Pomacea canaliculata* was selected as the outgroup species. Sequences were first aligned by the muscle v3.8.31 (Edgar 2004). The ModelFinder (Kalyaanamoorthy et al. 2017) was employed to calculate the best-fit model of nucleotide substitution for complete mitogenomes, and the best substitution model was TIM + F+R4 (BIC). A maximum likelihood (ML) tree was constructed by using IQ-TREE v 1.6.12 (Nguyen et al. 2015) with 1000 bootstrap replications (Figure 1). The ML tree showed two distinct clusters in the Cerithioidea superfamily, and *C. tonkiniana* was clustered with *Cerithidea sinensis* and *Cerithidea obtuse*, forming the Potamididae clade with 100% bootstrap support. This also showed the reliability of our assembled mitogenome of *C. tonkiniana.*

**Figure 1.**
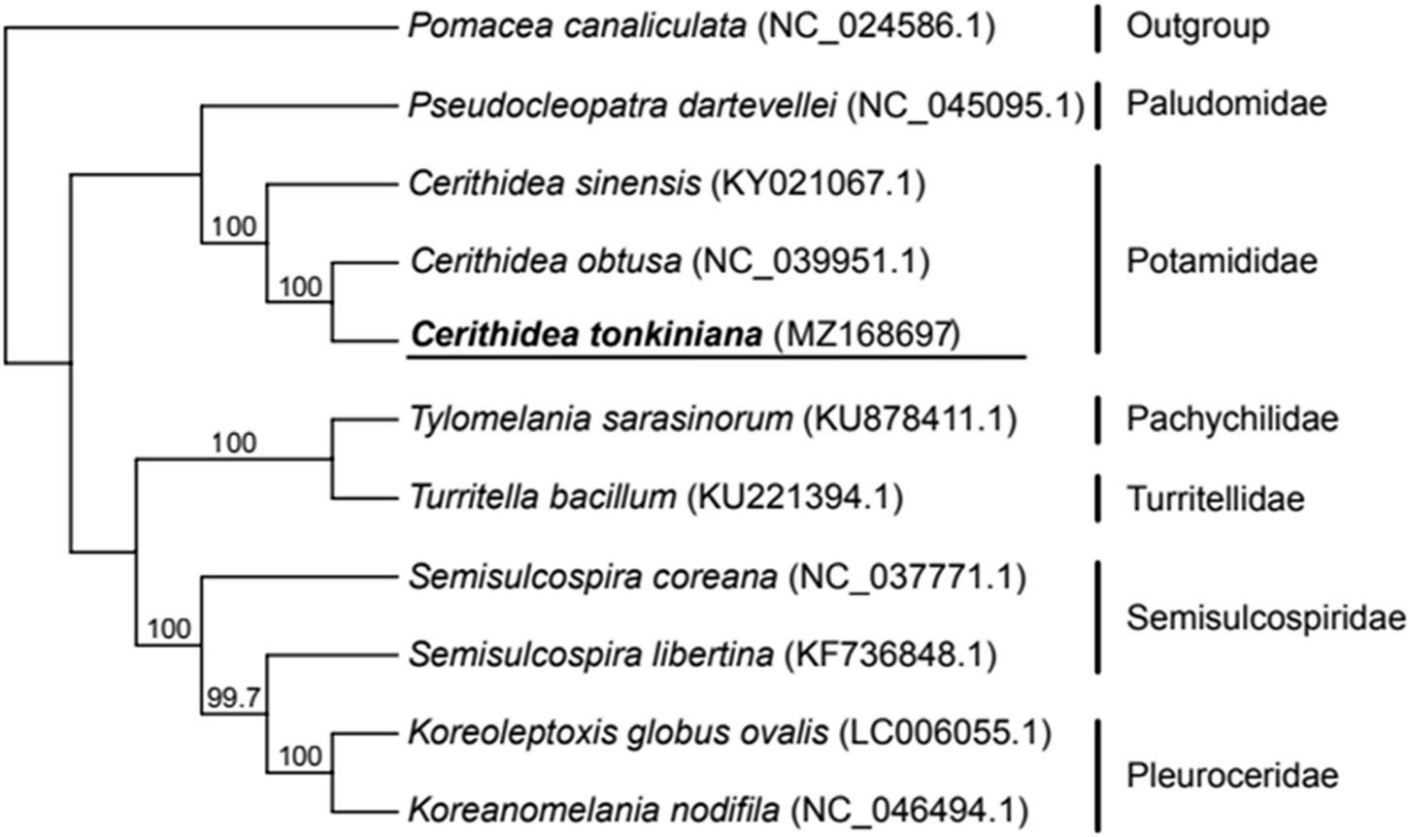
Maximum likelihood tree inferred by the complete mitochondrial genome of 10 Cerithioidea superfamily species.

## Data Availability

The assembled whole mitochondrial genome of *C. tonkiniana* is openly available in GenBank of NCBI website (http://www.ncbi.nlm.nih.gov) under the accession number of MZ168697. The associated BioProject and BioSample numbers are PRJNA742856, SAMN19981490, SRA for paired reads are SRR1531203.
